# Mutation screening in the genes *PAX-8, NKX2-5, TSH-R, HES-1* in cohort of 63 Brazilian children with thyroid dysgenesis

**DOI:** 10.20945/2359-3997000000065

**Published:** 2018-08-01

**Authors:** Taíse Lima de Oliveira Cerqueira, Yanne Rocha Ramos, Giorgia Bruna Strappa, Mariana Souza de Jesus, Jailciele Gonzaga Santos, Camila Sousa, Gildásio Carvalho, Vladimir Fernandes, Ney Boa-Sorte, Tatiana Amorim, Thiago Magalhães Silva, Ana Marice Teixeira Ladeia, Angelina Xavier Acosta, Helton Estrela Ramos

**Affiliations:** 1 Universidade Federal da Bahia Universidade Federal da Bahia Instituto de Ciências da Saúde Laboratório de Estudo da Tiroide Salvador BA Brasil Departamento de Biorregulação, Laboratório de Estudo da Tiroide, Instituto de Ciências da Saúde, Universidade Federal da Bahia (UFBA), Salvador, BA, Brasil; 2 Fundação Oswaldo Cruz Fiocruz Instituto Gonçalo Moniz Programa de Pós-Graduação em Biotecnologia em Saúde e Medicina Investigativa Salvador BA Brasil Programa de Pós-Graduação em Biotecnologia em Saúde e Medicina Investigativa, Instituto Gonçalo Moniz (IGM/ Fiocruz), Salvador, BA, Brasil; 3 Associação de Pais e Amigos dos Excepcionais Salvador BA Brasil Associação de Pais e Amigos dos Excepcionais (APAE), Salvador, BA, Brasil; 4 Grupo Fleury Salvador BA Brasil Grupo Fleury, Salvador, BA, Brasil; 5 Universidade Federal da Bahia Universidade Federal da Bahia Programa de Pós-Graduação em Saúde Pública Salvador BA Brasil Programa de Pós-Graduação em Saúde Pública, Universidade Federal da Bahia (UFBA), Salvador, BA, Brasil; 6 Escola Bahiana de Medicina e Saúde Pública Escola Bahiana de Saúde e Medicina Programa de Pós-Graduação em Saúde Humana e Medicina Salvador BA Brasil Programa de Pós-Graduação em Saúde Humana e Medicina, Escola Bahiana de Saúde e Medicina, Salvador, BA, Brasil

**Keywords:** Thyroid dysgenesis, congenital hypothyroidism, transcription factors

## Abstract

**Objective:**

To evaluate the candidate genes *PAX-8, NKX2-5, TSH-R* and *HES-1* in 63 confirmed cases of thyroid dysgenesis.

**Subjects and methods:**

Characterization of patients with congenital hypothyroidism into specific subtypes of thyroid dysgenesis with hormone levels (TT4 and TSH), thyroid ultrasound and scintigraphy. DNA was extracted from peripheral blood leukocytes and the genetic analysis was realized by investigating the presence of mutations in the transcription factor genes involved in thyroid development.

**Results:**

No mutations were detected in any of the candidate genes. *In situ* thyroid gland represented 71.1% of all cases of permanent primary congenital hypothyroidism, followed by hypoplasia (9.6%), ectopia (78%), hemiagenesis (6.0%) and agenesis (5.5%). The highest neonatal screening TSH levels were in the agenesis group (p < 0.001).

**Conclusions:**

Thyroid dysgenesis is possibly a polygenic disorder and epigenetic factors could to be implicated in these pathogeneses.

## INTRODUCTION

Congenital hypothyroidism (CH) is the most common disorder of the endocrine system among newborns with a global incidence of about 1/3,000-4,000 (1). Untreated CH can cause dwarfism and severe intellectual disability, with the delayed onset of thyroid replacement therapy by only a few weeks after birth being associated with reduced development of mental functions later on in life (2). Therefore, neonatal screening programs have been implemented to identify these patients earlier to ensure proper somatic growth and development of the central nervous system in infants (3).

The causes of CH are broadly categorized into dyshormonogenesis accounting for 15% of the cases, and thyroid dysgenesis (TD) accounting for 85% of the cases (3-6). Dyshormonogenesis is caused by autosomal recessive mutations of key molecules of thyroid hormone synthesis, in which thyroid hormone production fails in a structurally healthy thyroid gland (7). TD is caused by a wide range of different structural malformations in the thyroid that result in a wide variety of different phenotypes of CH (6,8-10). TD is subcategorized into: 1) thyroid agenesis - the most severe form, in which there is a complete lack of thyroid tissue (i.e. both lobes); 2) thyroid hemiagenesis - one of the thyroid lobes is completely missing; 3) thyroid hypoplasia - the gland is smaller but is still in a normal position; 4) thyroid ectopia - the gland is not positioned normally but rests along the migratory pathway of the primordium.

While TD appears sporadically in the vast majority of cases (8), several findings have pointed to a genetic underpinning (11-13). Familial inheritance patterns have been observed in about 2% of cases (14) and a higher incidence has been found for girls (male to female ratio 1:2) and among the Hispanic and Caucasian ethno-racial groups in comparison with Afro-descendants (15). Animal models have shown that several genes have been implicated from animal models were putatively associated with TD, however results from genetic association studies in human have been controversial (6).

The relevance of understanding the genetic underpinnings of TD has important implications not only for further understanding of the genotype/ phenotype relationship of the disorder, but also to assist in the correct management of patients and screening programs for CH.

The present study aimed investigate the etiology of the permanent CH diagnosed, with the purpose of identifying the cases due to TD, and evaluate the role of four different candidate genes that have been suggested as being involved in thyroid embryogenesis: (paired box gene 8 *(PAX-8),* thyroid stimulating hormone receptor *(TSH-R),* transcription factor related locus 5 *(NKX2-5)* and hairy/enhancer of split 1 *(HES-1)* and having an influence on these outcomes. We believe this study will contribute to the better knowledge of genetic basis of TD-caused CH.

## SUBJECTS AND METHODS

Patients with primary CH over the age of 3 years were recruited between 2012 and 2015 from the Association for Parents and Friends of Disabled Individuals (APAE) - Salvador - Bahia.

Retrospective analysis of patient medical records was conducted to evaluate the clinical presentation including: 1) TSH; total T4 (TT4), and free T4 (fT4) at the time of neonatal screening, measured by immunofluorometric assays (Autodelfia®, Wallac Oy, Turku, Finland); and 2) congenital heart defects or other congenital malformations.

The patients were characterized for TD by: 1) measuring thyroglobulin (Tg) levels, per immunofluorometric assays (Autodelfia®, Wallac Oy, Turku, Finland); 2) thyroid ultrasound, performed by Portable Mindray DP-4900 – 7-10 Mhz focused on the thyroid gland and cervical region. Total thyroid volume was calculated as described by Ueda, 1999 (16) and compared according to sex, age, height and body surface as described by Zimmermam and cols., 2004 (17); and 3) scintigraphy, performed by intravenous injection of *99Tc Pertechnetate.*

This study was approved by both the Federal University of Bahia - Ethics Committee for Research Projects and the APAE-Salvador (N° 125/2011 and N° 22/2011, respectively). Participation was voluntary with written consent obtained from at least one parent or guardian.

### Genetic testing

DNA was extracted from whole blood using PureLink® Genomic DNA Mini kit (Life Technology®, Carlsbad, California). The entire coding region and promoter region of the *PAX-8* gene, including exon/intron boundaries, was amplified from genomic DNA by polymerase chain reaction (PCR) using standard techniques (18). All 10 individual *TSH-R* exons were sequenced, with exon 10 subdivided into 2 overlapping primers (18). The coding region of the *NKX2-5* gene was studied and each of 2 exons were amplified by a total of 3 PCRs (18). *HES-1* exons were sequenced entirely using 5 pairs of primers with exon 4 divided into two parts (18). PCR products were sequenced directly on a ABI Prism® 3100 Genetic Analyzer (Applied Biosystem^®^, Carlsbad, California) using the Big Dye TM® Terminator Sequencing Standard Kit (Applied Biosystems®, Carlsbad, California). The results were analyzed by comparison with the standard sequence (www.ncbi.nlm.nih.gov/pubmed) for each gene specifically *(PAX-8; TSH-R; NKX2-5* and *HES-1)* using the bioinformatics program BioEdit Sequence Alignment Editor, Version 7.2.5.0 (Ibis Biosciences, Carlsbad, California). All primers and PCR conditions are available upon request.

### Statistical analysis

The clinical parameters (TSH and fT4 serum levels) of the patients diagnosed with TD were compared with those of patients diagnosed with ISTG to assess the severity of conditions in patients with different phenotypes. Statistical analysis was conducted with SPSS 2.0 and Stata 12 software packages. Nonparametric Mann-Whitney U and Kuskall-Wallis tests were used whenever appropriate and a p-value of < 0.05 was considered significant. The Dunn test was used for multiple comparisons (19).

## RESULTS

Of the 1,188 newborns diagnosed with CH up to the year 2016, abnormal TSH levels were confirmed in 773 newborns and they are being followed-up. Of these, 2.84% (N = 22) were found to be transitory primary congenital hypothyroidism, based upon spontaneous normalization of TSH between screening and diagnosis by the age of 3, thus resulting in 354 unrelated patients eligible for inclusion.

**Figure 1:**
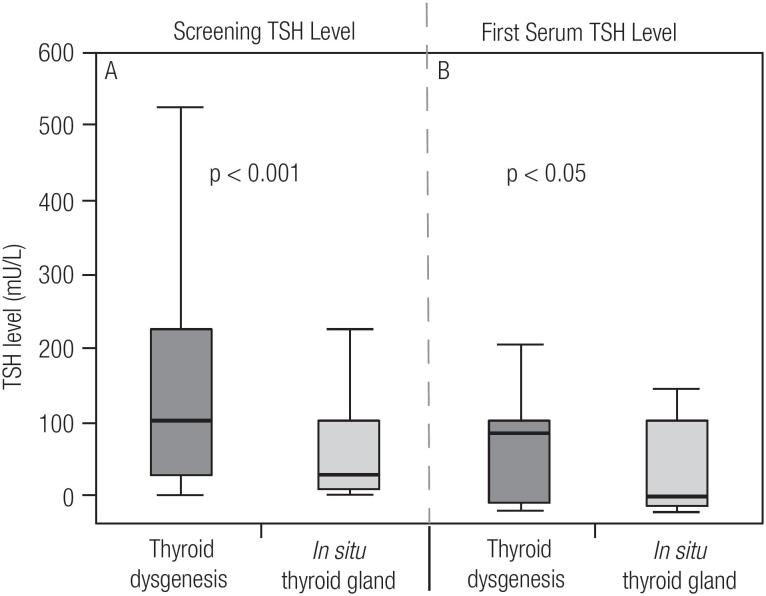
Comparison of median TSH levels of patients with thyroid dysgenesis and those with in situ thyroid gland. **A**) Comparison of Neonatal screening TSH levels **B**) Initial serum TSH. p-Value: Mann-Whitney U test.

**Figure 2:**
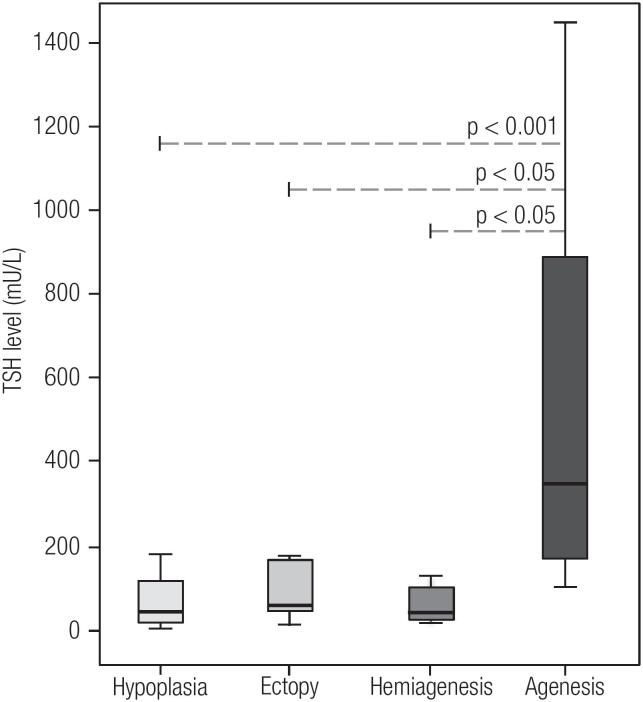
Comparison of screening TSH levels among the different subtypes of thyroid dysgenesis. P-Value: the Dunn test for multiple comparisons.

During the study period May 2012-June 2015, 218 patients underwent imaging tests for TD characterization. Of the 218, 155 (71.1%) had ISTG with the remaining 28.9% (N = 63) having some form of TD: ectopy (N = 17; 7.8%), agenesis (N = 12; 5.5%), hypoplasia (N = 21; 9.6%) and hemiagenesis (N = 13; 6.0%). The overall gender distribution was 118 females: 100 males. A summary of the clinical parameters evaluated in newborns is shown in Table 1. In a broader comparison, patients with TD compared with those with ISTG had significantly higher screening TSH levels (p < 0.001) and confirmatory serum TSH levels (p < 0.05) (Figure 1). This difference was probably due to the group of agenesis, as this group showed significantly higher levels than the other groups of TD (Figure 2). In addition, the agenesis subgroup had lower fT4 levels (0.4, IQR 0.3-1.2 μg/dL), followed by the ectopy group (0.8, IQR 0.3-1.1 μg/dL) when compared with the other groups.

**Table 1:** Newborn Screening Results by Diagnostic Category

N = 218	n (%)	Gender		Screening TSH (mU/L)	Confirmatory TSH (mU/L)	Serum fT4 (μg/dL)	Age of LT4 start (days)
		F	M	Median	Median	Median	Median
ISTG	155 (71.1)	77	78	31.3	23.5	0.95	18.5
Ectopy	17 (7.8)	14	3	53.6	95.0	0.8	15.5
Agenesis	12 (5.5)	10	2	344	100.0	0.4	27
Hypoplasia	21 (9.6)	11	10	39.4	26.5	1.0	29
Hemiagenesis	13 (6.0)	6	7	39.6	21.8	1.1	21

ISTG: *In situ* thyroid gland.

References values: Screening TSH: 9,0 mU/L. Confirmatory TSH: 0,3-4,0mU/L. Serun fT4: 0,9-2,6 ng/dL.

### Genetics findings

Six known single nucleotide polymorphisms (SNP) were found in patients with TD. Two in the *NKX2-5* gene: a) rs2277923 (g.173235021T>C) was found in 34/63 (54%) patients – 19 heterozygotes, 15 homozygotes; b) rs72554028 (g.173233001C>G) was found in one patient (1/63 – 2%). Four polymorphisms were found in the *TSH-R* gene: a) rs1991517 (g.81610583G>C) was found in 31/63 (49%) patients – 27 heterozygotes, 4 homozygotes; b) rs2234919 (g.81422178C>A) was found in 4/63 (6%) - all heterozygotes; c) rs752184247 (g.78929481G>A) was found in 14/63 (22%) - all heterozygotes and; d) rs200551849 (g.8589551C>T) was found in 2/63 (3%) patients - both compound heterozygotes with the rs1991517 polymorphism.

## DISCUSSION

None of the 63 patients with TD (41 females and 22 males) had mutations in the studied candidate genes *PAX-8, TSH-R, NKX2-5* and *HES-1.* The candidate genes *PAX-8, NKX2-5, TSH-R* and *HES-1* proposed as those being the most likely cause of TD (8,20-24) were not responsible for the TD confirmed in our cohort. This finding implies that there may be other genes that are responsible for TD (6-8). In the cases of the patients with agenesis and ectopia, there was a clear gender bias towards females that was not seen in the ISTG, hypoplasia or hemiagenesis groups, which had a virtually equal distribution between the two genders. Along with a greater gender discrepancy, those with ectopy (p ≤ 0.05) and agenesis (p ≤ 0.001) had medians of TSH that were notably higher at the initial newborn screening and confirmatory serum TSH testing compared with hypoplasia and hemiagenesis. To the best of our knowledge we are the first researchers in Bahia to perform this type of genetic analysis for candidate thyroid embryogenesis genes *PAX-8, NKX2-5, TSH-R* and *HES-1* in confirmed cases of permanent CH and TD (25). It should be noted that we excluded *NKX2-1* and *FOXE-1* from our analysis, based upon the fact that the phenotype presenting in our cohort showed no signs of Bamforth-Lazarus syndrome or neurological findings (26).

Overall, the incidence of CH found in the Brazilian neonatal screening is 1/2,595 to 1/4,795 (10,27) in accordance with the expected global incidence reported of 1/3,000-4,000 infants (3,6,9). ISTG was the most common etiology found in our cohort, with a rise in incidence from previously reported measures, primarily attributed to the diagnosis of milder cases of CH due to a lower TSH threshold used for screening (28).

The severity of hypothyroidism in children with TD has varied between studies. In our study, children with agenesis presented clinical parameters differing significantly from those of the other subtypes at both neonatal screening and confirmatory testing for hormone levels. This was consistent with the findings of other studies that have demonstrated that children with agenesis present worse neuropsychological development in comparison with children with hypoplasia, ectopy or dyshormonogenesis, and require higher doses of L-T4 (29-31). Thus, it is possible that treatment and followup schedules for TD need to consider the unique hormonal patterns and different responses to therapy in each different etiological category (4,7,30,31).

Similar to our study, only a few mutations have previously been found in large panels of patients (18,23). Mutations have previously been identified in familial groups with TD, however, in our population there are likely to be sporadic and unrelated cases, which could be an important factor to evaluate the impact of these genetic variants at population level (28). Of the polymorphisms found, rs1991517 in the *TSH-R* gene has frequently been reported in the Brazilian population (18,20,22) with a frequency as high as 10% (23), representing the second highest reported alteration in patients with CH. Previous studies, have found *PAX-8* mutations ranging from 0-3.4% (8). Inactivating mutations on the human *TSH-R* gene can cause a resistance to TSH with a strongly variable type of disease. Several studies have found that *TSH-R* mutations are more frequent than expected in patients with TD with prevalence in cohorts between 3-6% and even as high as 16.5% (8). *HES-1* has yet to be found in patients with TD (24). *NKX2-5,* also associated with cardiac malformations, was found with 3 heterozygote mutations in a single study with a cohort of 241 patients (32). In our study, seven patients (2 females and 5 males) presented congenital heart disease (CHD) associated with TD, previously described in electronic records (25).

Studies have hypothesized that the molecular mechanisms underway in the very early steps of thyroid organogenesis were underlying in the etiopathogenesis of TD (11,33,34). However, it continues to be difficult to draw many conclusions from the murine models, as many fundamental differences with human thyroid development have been reported (i.e. localization, mode of inheritance and penetrance) (33,35-38). For instance, while heterozygous *PAX-8* mutations in murine models do not display a pathological phenotype, heterozygous *PAX-8* mutations have been reported in sporadic and familial CH patients with thyroid hypoplasia or ectopy (35,39-41). However, a heterogeneous biochemical and morphological phenotype has been observed among patients with the same *PAX-8* mutations (40,41). At present, findings have suggested that genetic background plays an important role in phenotypic presentation (12,31,33,34). TD is likely to be of polygenic origin (12,31,34,36) and unlikely to occur from Mendelian transmission (12,31,34,37), with the existence of several modifier alleles contributing to a resulting phenotype (7,24,32,39,38,42). This is consistent with the theory that suggests that the molecular mechanisms resulting in defective thyroid morphogenesis may be “modulated by the genetic constitution of the embryo and/or the hormonal milieu of the fetus” (43). While many genes have been identified as important contributors to survival, proliferation and migration of thyroid precursor cells in fact, act as an integrated and complex regulatory network (7) and it will most probably require large samples of individuals to unravel the etiopathogenesis of TD.

One of the significant limitations of thyroid ultrasound is the poor sensitivity in visualizing the ectopic thyroid when compared with radionuclide scanning (22,44). In attempts to reduce the chances of misdiagnosis of TD etiology we included only those patients with a complete description of TD. Our genetic analysis was performed following a standard protocol, however, we did not perform any analysis of other modifications found in the promoter (except for the *PAX-8* gene) or complete intronic regions, which should also be considered.

In conclusion, we did not find any mutations in the proposed thyroid embryogenesis genes *TSH-R, PAX-8, NKX2-5,* and *HES-1* in 63 confirmed cases of TD. We found that patients with agenesis had clinically distinctive hormone levels at the time of the neonatal screening compared with those with other types of CH. The few genetic polymorphisms identified in the *TSH-R, PAX-8, NKX2-5,* and *HES-1* were not sufficient to elucidate the pathophysiology and the molecular mechanisms underlying defects in the cases of TD.
